# Three new species of the Homoneura (Homoneura) singularis group from China (Diptera, Lauxaniidae, *Homoneura*)

**DOI:** 10.3897/zookeys.1277.155340

**Published:** 2026-04-17

**Authors:** Peng-Yan You, Xu-Long Chen, Wen-Liang Li

**Affiliations:** 1 College of Horticulture and Plant Protection, Henan University of Science and Technology, Luoyang, Henan 471023, China Guizhou University Guiyang China; 2 Henan Province Engineering Technology Research Center of Green Plant Protection, Luoyang 471023, China Henan University of Science and Technology Luoyang China; 3 State Key Laboratory of Green Pesticides, Guizhou University, Guiyang, Guizhou 550025, China Henan Province Engineering Technology Research Center of Green Plant Protection Luoyang China

**Keywords:** Homoneurinae, species group, morphology, taxonomy, illustration

## Abstract

Three species of the subgenus Homoneura (Homoneura) Wulp, 1891 from China are described as new to science: H. (H.) furcata**sp. nov**., H. (H.) lishuiensis**sp. nov**., and H. (H.) sexangula**sp. nov**., which are assigned to the H. (H.) singularis group. A key to the species of this species group in China is presented. Detailed descriptions and illustrations of male genitalia of these new species are provided.

## Introduction

*Homoneura* Wulp, 1891, the most species-rich genus in the family Lauxaniidae, exhibits a near-cosmopolitan distribution, with the notable exception of the Neotropical region. There are five subgenera in China: *Chaetohomoneura* Malloch, 1927, *Euhomoneura* Malloch, 1927, *Homoneura* Wulp, 1891, *Minettioides* Malloch, 1929 and *Neohomoneura* Malloch, 1927 (Yang et al. 1999, 2001, 2002; Gao and Yang 2002, 2003; Gao et al. 2003; Shi and Yang 2008; Shi et al. 2011, 2012; Li and Yang 2015). Among these subgenera, H. (Homoneura) Wulp, 1891 is the most species-rich, with more than 700 described species from all zoogeographical regions except the Neotropical region. Notably, more than 220 species of this subgenus have been described from China. The subgenus *Homoneura* can be separated from other subgenera by the combination of the following characters: mesonotum with 0+3 dorsocentral setae, anteriormost dorsocentral seta behind transverse scutal suture, 0 supraalar seta, 0 intraalar seta, mid tibia without posterior seta.

The division of the subgenus *Homoneura* into different species groups is necessary to facilitate rapid identification. Prior to 2009, taxonomic classifications recognized 20 distinct species groups of the subgenus *Homoneura*, only three of which had been documented in the Chinese fauna (Kim 1994; Miller 1977; Papp 1978, 2006; Sasakawa 1992; Shi and Yang 2009). Shi and Yang (2009) first proposed 17 species groups of the subgenus *Homoneura* that were reported from China and later (Shi and Yang 2014) revised this classification of the Chinese fauna again, resulting in the current recognition of 21 species groups in the Chinese fauna. Recently, Yao et al. (2024) reconstructed the phylogeny of subgenus *Homoneura* using morphological data and proposed a revised classification comprising 12 species groups. However, due to insufficient supporting evidence for this revised framework, the present paper retains the conventional 21-group taxonomic system.

At present, there are 18 known species of *singularis* group in China (Becker 1895; Malloch 1927; Sasakawa and Ikeuchi 1985; Sasakawa 2001, 2002; Yang et al. 2002; Yang et al. 2003; Gao and Yang 2004; Shi and Yang 2014; Gao et al. 2016; Shen et al. 2018; Zhang et al. 2019). In this paper, three new species are described and assigned to the *singularis* group of the subgenus *Homoneura* Malloch by the wing with 2 brown spots between crossvein r-m and apical spot on R_4+5_. Until now, the number of species of *singularis* group is up to 21 species in China. The descriptions and illustrations of male genitalia of three new species are provided. A key to the species of this species group in China is also presented.

## Material and methods

General terminology follows Cumming and Wood (2017) and Gaimari and Miller (2021). Genitalia preparations were made by removing and macerating the apical portion of the abdomen in pancreatin for six hours (Álvarez-Padilla and Hormiga 2007), then rinsing them with distilled water for dissection and study. After examination in glycerin, they were transferred to an ethanol tube together with the wet specimens. Specimens examined were deposited in the Henan University of Science and Technology, Luoyang, Henan, China (**HAUST**).

## Taxonomy

**Family Lauxaniidae Macquart, 1835**

**Subfamily Homoneurinae Stuckenberg, 1971**

**Genus *Homoneura* Wulp, 1891**

**Subgenus *Homoneura* Wulp, 1891**

### Key to species of Homoneura (Homoneura) singularis group in China

Modified from Shi and Yang 2014; Zhang et al. 2019.

**Table:** 

1	Cell r_1_ with multiple brown spots (Figs 34–40)	**2**
–	Cell r_1_ with single brown spot apically (Figs 32, 33)	**8**
2	Brown spots of R_4+5_ between apical spot and brown cloud-like spot on crossvein r-m with hyaline rings (Figs 2, 21, 22, 38, 40)	**3**
–	Brown spots of R_4+5_ not as above	**4**
3	Surstylus (Figs 27, 28) curved and finger-like; syntergosternite 7+8 (Fig. 29) with 1 membranous triangular ventral process; hypandrium without ventral process	**H. (H.) sexangula sp. nov**.
–	Surstylus (Figs 6, 7) bifurcated apically; syntergosternite 7+8 (Fig. 8) semicircular; hypandrium with 1 long and bifurcated ventral process (Fig. 9)	**H. (H.) furcata sp. nov**.
4	Wing with 2 brown spots basally (Fig. 35), surstylus apical with 1 concavity between two acute processes (Sasakawa 2002: fig. 12)	**H. (H.) concava Sasakawa 2002**
–	Wing with 1 brown spot basally or only R_4+5_ vein basally brown (Figs 34, 36, 37, 39), surstylus not as above	**5**
5	Wing with 1 brown spot at base of vein R_4+5_, extending to base of cell r_2+3_ (Figs 34, 39)	**6**
–	Wing with only R_4+5_ vein basally brown (Figs 36, 37)	**7**
6	Fore femur with 7 posterior dorsal setae and 5 posterior ventral setae (Fig. 13), surstylus tapering apically in lateral view (Fig. 16)	**H. (H.) lishuiensis sp. nov**.
–	Fore femur with 6 posterior dorsal setae and 3 or 4 posterior ventral setae, surstylus apical truncation in lateral view (Gao et al. 2016: figs 1, 8–10)	**H. (H.) apicitrunctata Gao, Shi & Han, 2016**
7	Fore femur with 6 posterior dorsal setae, surstylus with a pair of spine-like processes in posterior view (Gao et al. 2016: figs 13, 19–23)	**H. (H.) dorsacerba Gao, Shi & Han, 2016**
–	Fore femur with 5 posterior dorsal setae, surstylus with 3 pairs of spine-like processes in posterior view (Gao et al. 2016: figs 14, 32–36)	**H. (H.) posterotricuspis Gao, Shi & Han, 2016**
8	Wing with 1 distinct brown spot at tip of R_1_	**9**
–	Wing without brown spot at tip of R_1_, at most subcostal cell dark apically	**14**
9	Wing with 1 brown spot at tip of R_1_ connected with a spot on crossvein r-m (Shi and Yang 2014: figs 334–335)	**H. (H.) zhejiangensis Shi & Yang, 2014**
–	Wing with 1 brown spot at tip of R_1_ separated from a spot on crossvein r-m	**10**
10	Parafacial with black inner margin	**11**
–	Parafacial without black inner margin	**13**
11	Abdomen black, more or less brown-tinged on lateral side, densely whitish gray dusted except for a median black fascia on tergites 2–5; male genitalia: surstylus knife-like with sparse short setulae and sharp in lateral view; hypandrium transverse bar-like (Sasakawa 2002: fig. 13)	**H. (H.) longicornis Sasakawa, 2002**
–	Abdomen yellow, tergites 2 or 3–6 with brown or black medial stripes and a pair of lateral spots; surstylus and hypandrium not as above	**12**
12	Ctenidium on fore femur with 14 short setae; antenna yellow except for 1^st^ flagellomere black on dorsal half; surstylus concaved backward and blunt at apex; phallus with a pair of furcated lateral processes at middle in ventral view (Shi and Yang 2014: figs 113–115)	**H. (H.) fengyangshanica Shi & Yang, 2014**
–	Ctenidium on fore femur with 16–17 short setae; antenna entirely yellow; surstylus extending downward; phallus with a pair of sharp apical processes in ventral view (Zhang et al. 2019: figs 22–32)	**H. (H.) dorsocuspidata Gao & Shi, 2019**
13	Wing with 1 brown preapical spot on M_1_, but without stripe-like spot on apical half of CuA_1_; surstylus originated from ventral margin of epandrium, broad knife-like in lateral view; postgonite short and wide, apically acute (Sasakawa and Ikeuchi 1985: 497: figs 5A, B)	**H. (H.) aulatheca Sasakawa & Ikeuchi, 1985**
–	Wing with 1 brown apical spot and 1 narrow subapical spot on apical 1/2 of M_1_, and 1 brown thin transverse stripe-like spot on apical half of CuA_1_; surstylus originated from inside of epandrium, falciform in lateral view; postgonite slender, apically blunt (Shi and Yang 2014: figs 107, 110)	**H. (H.) falcata Shi & Yang, 2014**
14	Mesonotum without brown stripe; abdomen without spot	**15**
–	Mesonotum with 2–6 brown stripes; abdomen with spot	**17**
15	Wing with brown spots on R_4+5_ and M_1_ separated; surstylus not as below	**16**
–	Wing with brown spots on R_4+5_ and M_1_ confluent, forming a large brown area between two apical spots; surstylus curved and constricted at middle (Sasakawa and Ikeuchi 1982: fig. 7)	**H. (H.) latifrons Malloch, 1927**
16	Abdomen dark brown, surstylus consisting of a small triangular anterior ventral process with setulae and a long digitiform apical process with a tiny middle tooth and acute tip in lateral view (Gao and Yang 2004: fig. 44)	**H. (H.) tianlinensis Gao & Yang, 2004**
–	Abdomen pale-yellow; surstylus with claviform single process (Shen et al. 2018: fig. 54)	**H. (H.) lamellata (Becker, 1895)**
17	Mesonotum with 4–6 brown stripes; epandrium with a blunt triangular subapical process in lateral view	**18**
–	Mesonotum with 2 brown stripes; epandrium straight on posterior margin, without triangular subapical process in lateral view; surstylus claviform, bulged at middle in lateral view	**19**
18	Arista pubescent, with longest ray shorter than 1/4 height of 1^st^ flagellomere; mesonotum with 4 brown medial stripes and 2 postsutural lateral stripes; hypandrium broad, hypandrial apodeme long; phallus with a pair of crossed subuliform dorsal processes in ventral view (Shi and Yang 2014: fig. 298)	**H. (H.) subvittata Malloch, 1927**
–	Arista short plumose, with longest ray as long as 1/2 height of 1^st^ flagellomere; mesonotum with 4 brown medial stripes; hypandrium narrow, hypandrial apodeme short; phallus without crossed subuliform dorsal processes in ventral view (Yang et al. 2003: figs 29–800B, 29–800C)	**H. (H.) didyma Yang, Hu & Zhu, 2003**
19	Ctenidium on fore femur with 15 or 16 short setae; surstylus long claviform, extended ventrally and acute apically with a small ventral process (Sasakawa 2001: fig. 26B)	**H. (H.) vittigera Sasakawa, 2001**
–	Ctenidium on fore femur with 11 or 12 short setae; surstylus not as above	**20**
20	Surstylus curved as a sickle with an acute apical tooth, several small teeth on dorsal margin and long setulae on dorsal and ventral margins; phallus narrow with a wide and deep apical concavity in ventral view, and apical margin broad and lateral sclerite turned outside apically in lateral view (Shi and Yang 2014: figs 133, 136, 137)	**H. (H.) hongmaoensis Shi & Yang, 2014**
–	Surstylus straight claviform, acute apically in lateral view; phallus with truncate apically in ventral view (Yang et al. 2002: figs 11, 12)	**H. (H.) singularis Yang, Hu & Zhu, 2002**

#### Homoneura (Homoneura) furcata sp. nov.

Taxon classificationAnimaliaDipteraLauxaniidae

704F30C0-BF86-5AB2-8A71-6944BF3D5A3A

https://zoobank.org/40594F67-65E6-46AA-89E6-ABB07BB3EC65

Figs 1, 2, 3, 4, 5, 6, 7, 8, 9, 10

##### Chinese name.

端叉同脉缟蝇

##### Type material.

***Holotype***: China • ♂; Chongqing City, Wuxi County, Yintiaoling Nature Reserve, Yezhuping; 31°29'45"N, 109°43'25"E; 2385 m elev.; 22.VI.2022; leg. Xulong Chen; HAUST.

***Paratypes***: China • 1♂4♀♀; Chongqing City, Wuxi County, Yintiaoling Nature Reserve, Lanying Town, Huangcaoping; 31°25'10"N, 109°55'43"E; 2104 m elev.; 30.VI.2022; leg. Xulong Chen; HAUST. • 1♂; Chongqing City, Wuxi County, Yintiaoling Nature Reserve, Lanying Town, Huangcaoping; 31°24'58.39"N, 109°55'24.78"E; 2039 m elev.; 14.VIII.2022; leg. Xulong Chen; HAUST. • 1♂1♀; Chongqing City, Wuxi County, Yintiaoling Nature Reserve, Guanshan forest farm, Shiyazi; 31°32'12"N, 109°41'58"E; 2145 m elev.; 21.VI.2022; leg. Xulong Chen; HAUST. • 2♂♂6♀♀; Hubei Prov., Shennongjia Forestry District, Shennong peak; 31°26'31.02"N, 110°07'02.96"E; 1558 m elev.; 26.VIII.2022; leg. Siqi Wang & Bintao Du; HAUST.

##### Etymology.

Latin, *furcata*, refers to the bifurcated ventral process of the hypandrium. A feminine adjective.

##### Diagnosis.

Wing with 1 brown spot at tip of subcostal cell, with 1 brown round spot at base of R_4+5_; cell r_1_ with 2 or 3 brown spots, 1 large brown round spot with 4 hyaline spots on crossvein r-m, 1 large brown spot with 3 hyaline spots on crossvein dm-cu; syntergosternite 7+8 semicircular; surstylus bifurcated apically in lateral view; hypandrium with a long, bifurcated ventral process apically.

##### Description.

**Male**. Body length 3.1–4.3 mm; wing length 3.9–4.3 mm.

**Figures 1–5.**
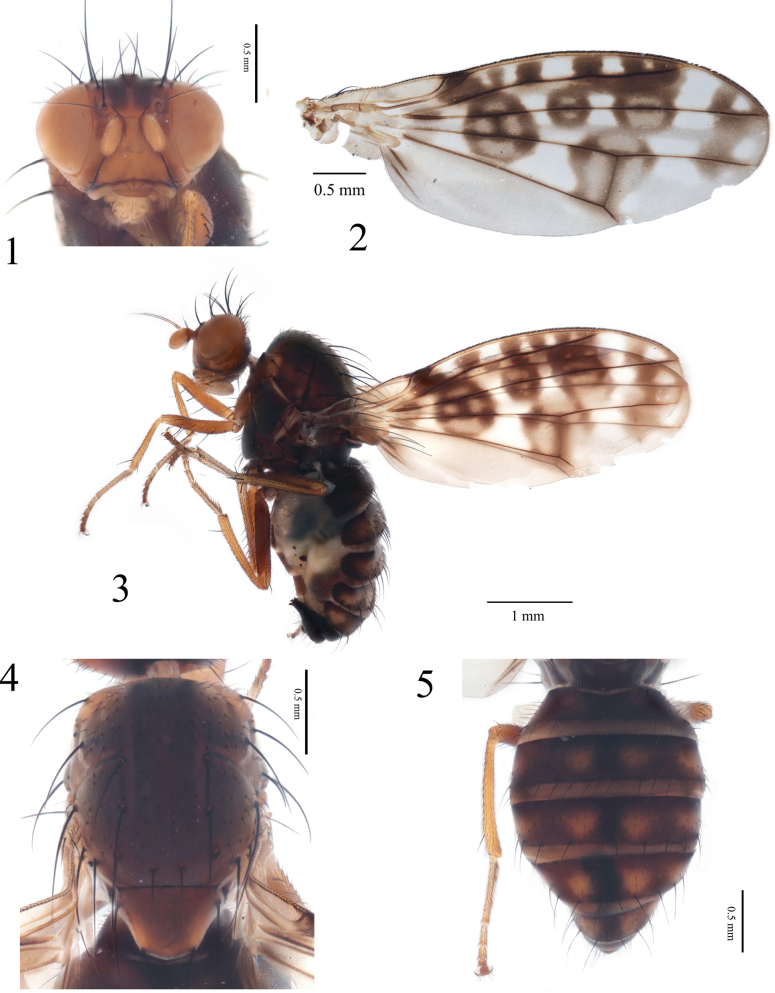
Homoneura (Homoneura) furcata sp. nov., male. **1**. Head, anterior view; **2**. Wing; **3**. Habitus, lateral view; **4**. Thorax, dorsal view; **5**. Abdomen, dorsal view.

***Head*** (Fig. 1) brownish yellow, with grey pruinosity. Parafacial with brown inner margin and a brown spot between eye and base of antenna. Frons as long as wide and parallel-sided, with a pair of brown longitudinal stripes extending to ocellar triangle; ocellar triangle blackish brown; ocellar setae developed, slightly longer than anterior fronto-orbital seta; anterior fronto-orbital seta shorter than posterior fronto-orbital seta. Gena about 1/6 height of eye. Antenna yellow, first flagellomere about 1.5 times longer than high; arista black except brown at base, pubescent, with longest ray shorter than 1/7 height of first flagellomere. Prementum and labellum yellow, with pale-yellow and black setulae; palpus pale-brown, with black setulae.

***Thorax*** (Fig. 4) blackish brown, with grey pruinosity. Dorsocentral setae 0+3; acrostichal setulae in 6 rows, short, hair-like; a pair of prescutellar setae, shorter than anteriormost dorsocentral setae. ***Legs*** mostly yellow; femur brown. Fore femur with 5 or 6 posterior dorsal setae, 4 posterior ventral setae, and ctenidium with 7–9 short setae; fore tibia with 1 strong dorsal preapical seta and 1 short apical ventral seta. Mid femur with 5 or 6 anterior setae and 1 short apical posterior seta; mid tibia with 1 strong dorsal preapical seta and 2 strong apical ventral setae. Hind femur with 1 preapical anterior dorsal seta; hind tibia with 1 long dorsal preapical seta and 1 short apical ventral seta. ***Wing*** (Fig. 2) yellow, with 1 brown irregular apical spot on R_2+3_; tip of subcostal cell with 1 brown spot; cell r_1_ with 2 or 3 brown spots; R_4+5_ with 1 brown round spot at base, 2 round or hexagonal brown spots with hyaline rings between crossvein r-m and apical spot; brown, irregular apical spots on R_4+5_ and M_1_ confluent; 1 large brown round spot with 4 hyaline spots on crossvein r-m; 1 large brown spot with 3 hyaline spots on crossvein dm-cu. Costa with 2^nd^ (between R_1_ and R_2+3_), 3^rd^ (between R_2+3_ and R_4+5_), and 4^th^ (between R_4+5_ and M_1_) sections in proportion of 4.0: 2.0: 1.0; crossvein r-m distinctly before middle of discal cell; ultimate and penultimate sections of M_1_ in proportion of 1.2:1.0; ultimate section of CuA_1_ about 1/10 of penultimate. Haltere pale-yellow.

**Figures 6–10.**
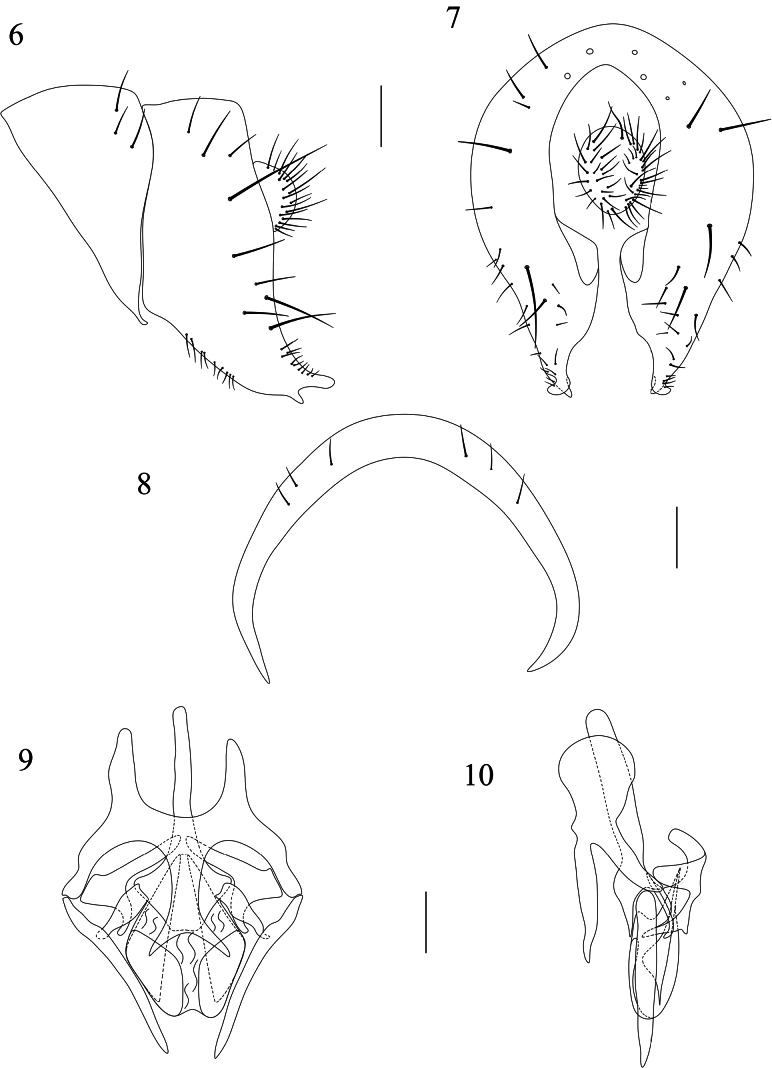
Homoneura (Homoneura) furcata sp. nov., male. **6**. Syntergosternite 7+8 and epandrial complex, lateral view; **7**. Epandrial complex, posterior view; **8**. Syntergosternite 7+8, anterior view; **9**. Phallic complex, ventral view; **10**. Phallic complex, lateral view. Scale bars: 0.1 mm.

***Abdomen*** (Fig. 5) brown, with grey pruinosity; tergites 2–6 each with a blackish brown medial spot and 2 pairs of yellow-brownish round lateral spots. ***Male genitalia*** (Figs 6–10): syntergosternite 7+8 semicircular; surstylus bifurcated apically in lateral view. Hypandrium H-shaped and with a long and bifurcated ventral process. Pregonite long, spine-like; postgonite curved. Phallus apical without concavity; phallapodeme longer than phallus.

**Female**. Body length 3.6–4.4 mm; wing length 4.2–4.4 mm. Sternite VIII triangular and wider than long, with long setae on lateral margin. Sternite IX V-shaped and concave on anterior margin.

##### Distribution.

China (Chongqing, Hubei).

##### Remarks.

This new species is similar to Homoneura (H.) nubecula Sasakawa, 2001 in wing spots, but it can be separated from the latter by the following: thorax without extensive irregular brown spot or band, surstylus bifurcated apically in lateral view, and hypandrium H-shaped and with a long and bifurcated ventral process. In H. (H.) nubecula, the thorax has numerous irregular brown spots or bands, and there are brown spots at the bases of the setae.

It is also similar to Homoneura (H.) sexangula sp. nov. in having 2 round or hexagonal brown spots with hyaline rings between crossvein r-m and apical spot on the wing, but in H. (H.) sexangula sp. nov., the syntergosternite is circular with 1 ventral process, and the hypandrium is without ventral process.

#### Homoneura (Homoneura) lishuiensis sp. nov.

Taxon classificationAnimaliaDipteraLauxaniidae

6E45EDB3-F640-5B95-A807-2E367691EAF7

https://zoobank.org/FD264371-489F-46F4-BB1A-1F5580C878B8

Figs 11, 12, 13, 14, 15, 16, 17, 18, 19, 20

##### Chinese name.

丽水同脉缟蝇

##### Type material.

***Holotype***: China • ♂; Zhejiang Prov., Lishui City, Fengyang Mountain, Fengyangjian; 27°54'02"N, 119°09'34"E; 1664 m elev.; 1.V.2021; leg. Xulong Chen; HAUST.

***Paratypes***: China • 4♂♂4♀♀; same data as holotype; HAUST. • 4♂♂3♀♀; Zhejiang Prov., Lishui City, Fengyang Mountain, Huangmaojian; 27°53'38"N, 119°10'05"E; 1515 m elev.; 30.IV.2021; leg. Zhaoyang Kong; HAUST. • 2♂♂1♀; Hunan Prov., Shaoyang City, Chengbu County, Jintong Mountain; 26°08'34"N, 110°12'53"E; 1103 m elev.; 23.VIII.2020; leg. Xulong Chen; HAUST. • 3♂♂; Guangdong Prov., Shaoguan City, Nanling Nature Reserve; 1880 m elev.; 22.VII.2020; leg. Wei Zeng; HAUST. • 4♂♂8♀♀; Guangxi Prov., Guilin City, Xing’an County, Maoer Mountain; 25°54'58"N, 110°27'57"E; 1735 m elev.; 30.VIII.2020; leg. Xulong Chen; HAUST. • 3♂♂4♀♀; Guangxi Prov., Guilin City, Xing’an County, Maoer Mountain; 25°54'31"N, 110°28'15"E; 1551 m elev.; 5.VI.2023; leg. Pengyan You; HAUST. • 2♂♂1♀; Guangxi Prov., Guilin City, Huaping National Nature Reserve; 25°33'21"N, 109°56'38"E; 1340 m elev.; 2.VI.2023; leg. Pengyan You; HAUST.

##### Etymology.

The specific epithet is named for the type locality, Lishui City.

##### Diagnosis.

Face with a pair of pale-brown median stripes; acrostichal setulae irregular in 6 rows; wing with brown spot at subcostal cell and brown spot on crossvein r-m confluent and forming a brown stripe. Surstylus tapering apically in lateral view; postgonite claviform. Phallus with indistinct concavity apically, round apically in lateral view.

##### Description.

**Male**. Body length 4.2–4.5 mm; wing length 4.3 mm.

**Figures 11–15.**
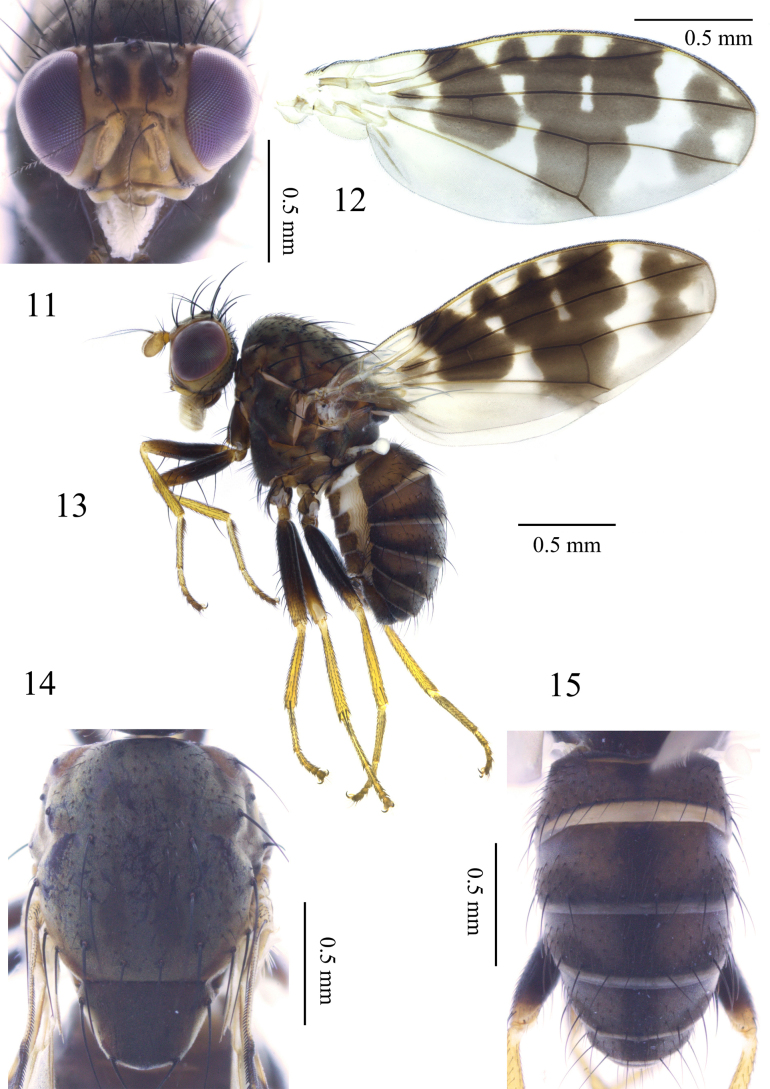
Homoneura (Homoneura) lishuiensis sp. nov., male. **11**. Head, anterior view; **12**. Wing; **13**. Habitus, lateral view; **14**. Thorax, dorsal view; **15**. Abdomen, dorsal view.

***Head*** (Fig. 11) brownish yellow. Face with a pair of pale-brown median stripes; parafacial with brown inner margin, with a pale-brown spot between eye and base of antenna. Frons as long as wide and parallel-sided, with a pair of brown longitudinal stripes extending to ocellar triangle; ocellar triangle blackish brown; ocellar setae developed, as long as anterior fronto-orbital seta; anterior fronto-orbital seta slightly shorter than posterior fronto-orbital seta. Gena about 1/6 height of eye. Antenna yellow, first flagellomere about 1.6 times longer than high; arista brown except yellow at base, pubescent, with longest ray shorter than 1/6 height of first flagellomere. Prementum and labellum yellow, with pale-yellow and black setulae; palpus pale-brown, with black setulae.

***Thorax*** (Fig. 14) blackish brown, with grey pruinosity. Dorsocentral setae 0+3; acrostichal setulae irregular in 6 rows, short hair-like; a pair of prescutellar setae, shorter than anteriormost dorsocentral setae. ***Legs*** mostly yellow; femur brown except yellow on apical 1/3. Fore femur with 7 posterior dorsal setae, 5 posterior ventral setae, and ctenidium with 12 short setae; fore tibia with 1 dorsal preapical seta and 1 short apical ventral seta. Mid femur with 5 or 6 anterior setae and 1 short apical posterior seta; mid tibia with 1 strong dorsal preapical seta and 3 strong apical ventral setae. Hind femur with 1 preapical anterior dorsal seta; hind tibia with 1 long dorsal preapical seta and 1 short apical ventral seta. ***Wing*** (Fig. 12) slightly yellow, with 1 large brown irregular apical spot and 1 brown irregular median spot on R_2+3_; brown spot at subcostal cell and brown spot on crossvein r-m confluent and forming a brown stripe. Wing with 1 small brown round spot at base of R_4+5_; 2 brown spots present between brown stripe on crossvein r-m and brown apical spot on R_4+5_, confluent with 2 irregular brown spots on R_2+3_ and brown cloud-like spot on crossvein dm-cu; irregular brown preapical spot on M_1_ confluent with brown apical spot on R_4+5_. Costa with 2^nd^ (between R_1_ and R_2+3_), 3^rd^ (between R_2+3_ and R_4+5_), and 4^th^ (between R_4+5_ and M_1_) sections in proportion of 6.0: 3.0: 1.6; crossvein r-m distinctly before middle of discal cell; ultimate and penultimate sections of M_1_ in proportion of 1.3:1.0; ultimate section of CuA_1_ about 1/8 of penultimate. Haltere pale-yellow.

**Figures 16–20.**
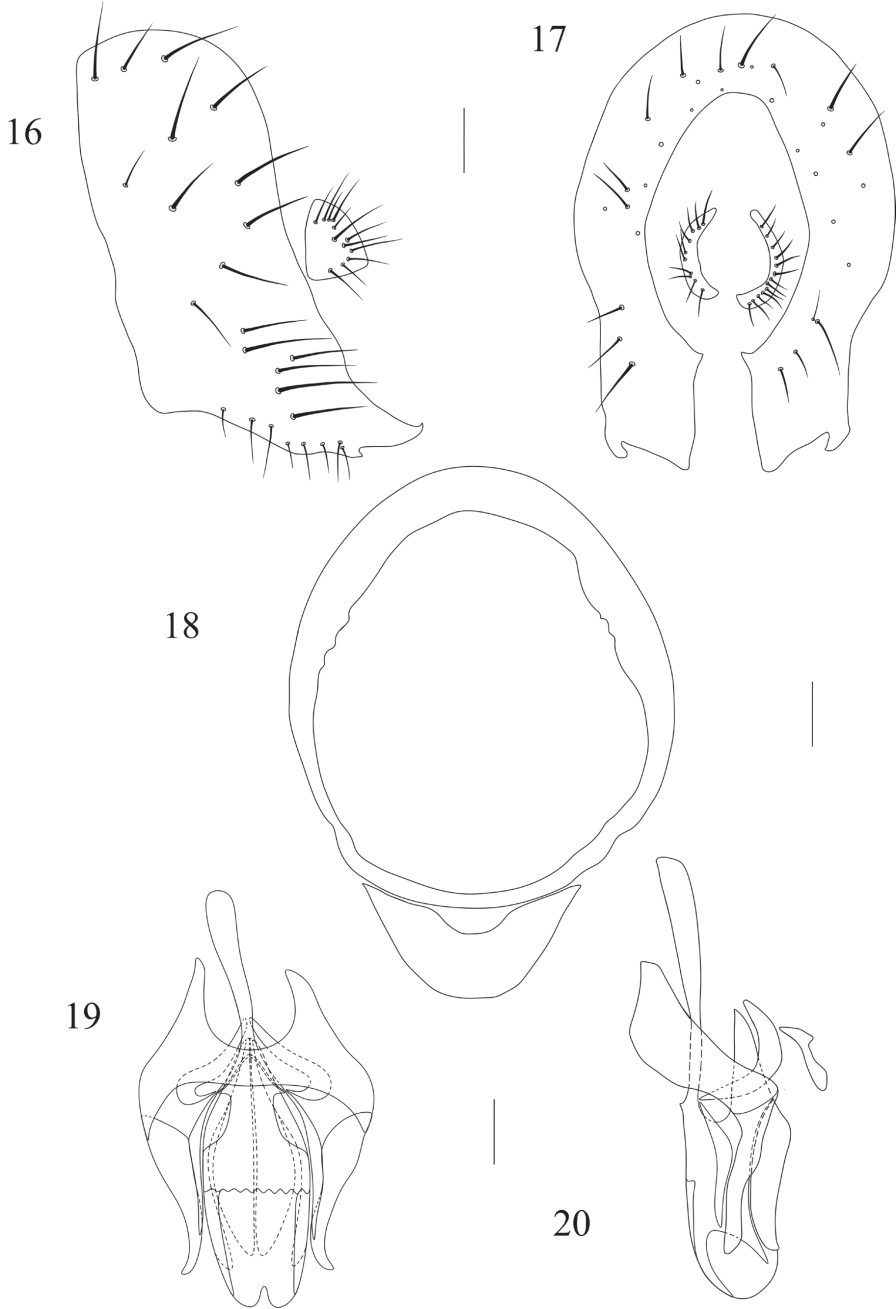
Homoneura (Homoneura) lishuiensis sp. nov., male. **16**. Epandrial complex, lateral view; **17**. Epandrial complex, posterior view; **18**. Syntergosternite 7+8, anterior view; **19**. Phallic complex, ventral view; **20**. Phallic complex, lateral view. Scale bars: 0.1 mm.

***Abdomen*** (Fig. 15) brown, with grey pruinosity; tergites 2–6 each with 1 blackish brown medial spot and a pair of blackish brown triangular lateral spots. ***Male genitalia*** (Figs 16–20): syntergosternite 7+8 circular with 1 membranous crescent-like ventral process; epandrium broad in lateral view. Surstylus tapering apically in lateral view. Hypandrium H-shaped. Pregonite claviform. Phallus with indistinct concavity apically, round apically in lateral view; phallapodeme as long as phallus.

**Female**. Body length 4.4–4.6 mm; wing length 4.0–4.2 mm. Sternite VIII significantly longer than wide, with a broad posterior margin and a narrow, slightly pointed anterior margin.

##### Distribution.

China (Zhejiang, Hunan, Guangxi, Guangdong)

##### Remarks.

This new species is somewhat similar to Homoneura (H.) concava Sasakawa, 2002 in wing patterning, but it can be separated from it by the surstylus tapering apically in lateral view, hypandrium H-shaped, and phallus with indistinct concavity apically. In H. (H.) concava, the surstylus is short, with a concavity, the hypandrium is transverse, with very short, lateral apodeme, and the phallus has a distinct apical concavity.

It is also similar to Homoneura (H.) apicitrunctata Gao, Shi & Han, 2016 in the form of the surstylus, but it can be separated from it by the following: mesonotum without median stripe and lateral stripe; brown median spots on R_4+5_ entirely confluent with 1 brown cloud-like spot on crossvein dm-cu; syntergosternite 7+8 circular with 1 ventral process; surstylus tapering apically in lateral view; hypandrium without ventral process; phallus without teeth-like process. In H. (H.) apicitrunctata, the syntergosternite 7+8 is semicircular and without ventral process; the surstylus is apically truncate; the hypandrium has 1 acute ventral process; and the phallus bears a pair of tooth-like processes (Gao et al. 2016: figs 4, 7, 8, 9, 11, 12).

#### Homoneura (Homoneura) sexangula sp. nov.

Taxon classificationAnimaliaDipteraLauxaniidae

9EA26640-E561-54A2-B104-C9A330A6D786

https://zoobank.org/7EF70E96-8490-403A-8DE4-36BE35BB170A

Figs 21, 22, 23, 24, 25, 26, 27, 28, 29, 30, 31

##### Chinese name.

六边同脉缟蝇

##### Type material.

***Holotype***: China • ♂; Sichuan Prov., Liangshan State, Mianning County, Yele Nature Reserve; 28°55'03"N, 102°11'46"E; 2662 m elev.; 10.VII.2019; leg. Zhudong Liu & Zhiming Li; HAUST.

***Paratypes***: China • 1♂2♀♀; same data as holotype; HAUST. • 3♂♂4♀♀; Chongqing City, Wuxi County, Yintiaoling Nature Reserve, Guanshan forest farm; 31°31'08"N, 109°43'01"E; 1984 m elev.; 21.VI.2022; leg. Xulong Chen; HAUST. • 1♂2♀♀; Chongqing City, Wuxi County, Yintiaoling Nature Reserve, Guanshan Forest Farm, Suokouyan; 31°28'38"N, 109°45'59"E; 1904 m elev.; 19.VIII.2022; leg. Xulong Chen; HAUST. • 1♂; Chongqing City, Wuxi County, Yintiaoling Nature Reserve, Lanying Town, Huangcaoping; 31°24'58"N, 109°55'25"E; 2039 m elev.; 14.VIII.2022; leg. Xulong Chen; HAUST. • 2♀♀; Chongqing City, Wuxi County, Yintiaoling Nature Reserve, Tianchiba; 31°31'07"N, 109°42'59"E; 1967 m elev.; 24.VI.2022; leg. Xulong Chen; HAUST. • 2♂♂3♀♀; Chongqing City, Wuxi County, Yintiaoling Nature Reserve, Yezhuping; 31°29'45"N, 109°43'25"E; 2385 m elev.; 22.VI.2022; leg. Xulong Chen; HAUST. • 4♂♂8♀♀; Chongqing City, Wuxi County, Yintiaoling Nature Reserve, Liaowangtai; 31°30'08"N, 109°42'25"E; 2524 m elev.; 22.VI.2022; leg. Xulong Chen; HAUST. • 4♂♂; Chongqing City, Wuxi County, Yintiaoling Nature Reserve, Guanshan forest farm, Shiyazi; 31°32'12"N, 109°41'58"E; 2145 m elev.; 21.VI.2022; leg. Xulong Chen; HAUST. • 1♂; Chongqing City, Wuxi County, Yintiaoling Nature Reserve, Lanying Town, Huangcaoping; 31°24'58"N, 109°55'24"E; 2043 m elev.; 29.VI.2022 (L); leg. Xulong Chen; HAUST. • 2♀♀; Chongqing City, Wuxi County, Yintiaoling Nature Reserve, Benzhuping; 31°29'22"N, 109°47'37"E; 1794 m elev.; 26.VI.2022; leg. Xulong Chen; HAUST.

##### Etymology.

Latin, *sexangula* (“six angled”, “hexagonal”) refers to the two hexagonal annular spots on R_4+5_. A feminine adjective.

##### Diagnosis.

Thorax with anteriormost postsutural dorsocentral seta on transverse suture of scutum; wing with 2 brown spots with hyaline rings on R_4+5_. Fore femur with 6 posterior dorsal setae and ctenidium with 6 or 7 short setae. Syntergosternite 7+8 circular with 1 membranous triangular ventral process. Pregonite slender, finger-like; postgonite slender coniform; phallus curved in lateral view.

##### Description.

**Male**. Body length 4.0–4.1 mm; wing length 4.1–4.3 mm.

***Head*** (Fig. 23) brownish yellow. Face with a brown median stripe; parafacial brown on inner margin, with a brown spot between eye and base of antenna. Frons wider than long and parallel-sided, with 2 brown longitudinal stripes extending to ocellar triangle; ocellar triangle black; ocellar setae developed, longer than anterior fronto-orbital seta; anterior fronto-orbital seta slightly shorter than posterior fronto-orbital seta. Gena about 1/5 height of eye. Antenna yellow, first flagellomere about 1.5 times longer than high; arista brown except yellow at base, pubescent, with longest ray shorter than 1/6 height of first flagellomere. Prementum and labellum yellow, with pale-yellow and black setulae; palpus yellow, with black setulae.

**Figures 21–26.**
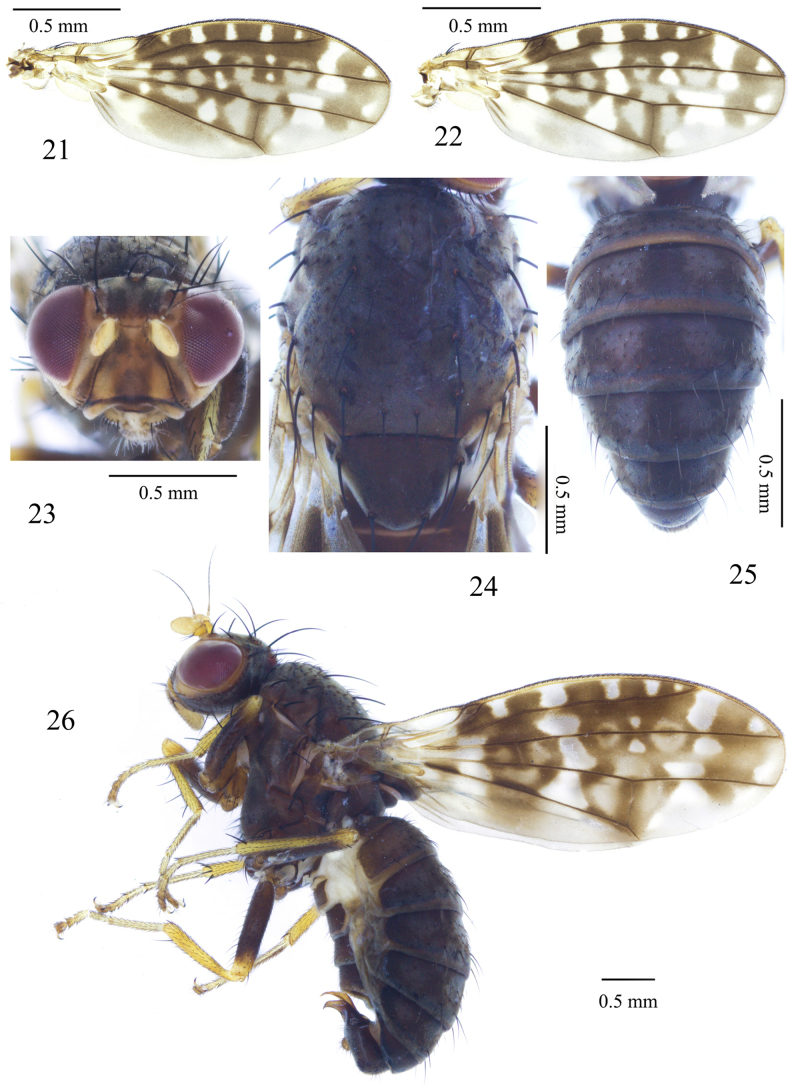
Homoneura (Homoneura) sexangula sp. nov., male. **21, 22**. Wing; **23**. Head, anterior view; **24**. Thorax, dorsal view; **25**. Abdomen, dorsal view; **26**. Habitus, lateral view.

***Thorax*** (Fig. 24) blackish brown, with grey pruinosity. Dorsocentral setae 0+3; anteriormost postsutural dorsocentral seta on scutal suture; acrostichal setulae in 6 rows, short hair-like; a pair of prescutellar setae, shorter than anteriormost dorsocentral setae. ***Legs*** mostly yellow; femur brown except yellow on apical 1/5. Fore femur with 6 posterior dorsal setae, 5 posterior ventral setae, and ctenidium with 6 or 7 short setae; fore tibia with 1 dorsal preapical seta and 1 apical ventral seta. Mid femur with 5 or 6 anterior setae and 1 short apical posterior seta; mid tibia with 1 strong dorsal preapical seta and 2 strong apical ventral setae. Hind femur with 1 preapical anterior dorsal seta; hind tibia with 1 long dorsal preapical seta and 1 short apical ventral seta. ***Wing*** (Figs 21, 22) with 1 irregular apical spot and 2 brown spots with hyaline rings on R_4+5_; brown irregular preapical spot on M_1_ confluent with brown apical spot on R_4+5_; cell r_1_ brown with several hyaline spots. Crossveins r-m and dm-cu each with 1 irregular annular spot. Wing with brown spot at base of R_4+5_, M_1_ and CuA_1_; costa with 2^nd^ (between R_1_ and R_2+3_), 3^rd^ (between R_2+3_ and R_4+5_), and 4^th^ (between R_4+5_ and M_1_) sections in proportion of 6.0: 2.7: 1.5; crossvein r-m distinctly before middle of discal cell; ultimate and penultimate sections of M_1_ in proportion of 1.4:1.0; ultimate section of CuA_1_ about 1/8 of penultimate. Haltere pale-yellow.

**Figures 27–31.**
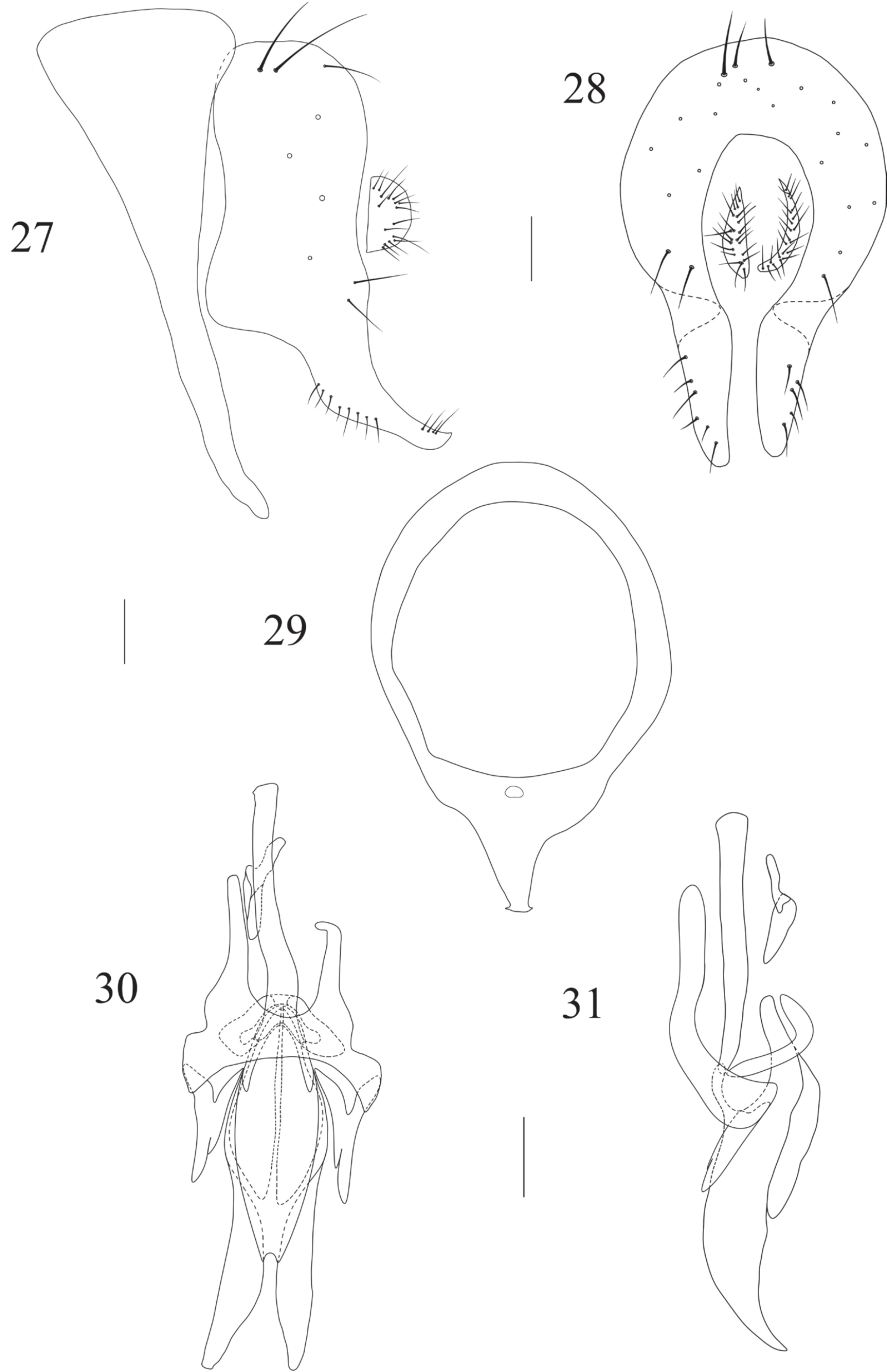
Homoneura (Homoneura) sexangula sp. nov., male. **27**. Syntergosternite 7+8 and epandrial complex, lateral view; **28**. Epandrial complex, posterior view; **29**. Syntergosternite 7+8, anterior view; **30**. Phallic complex, ventral view; **31**. Phallic complex, lateral view. Scale bars: 0.1 mm.

***Abdomen*** (Fig. 25) brown, with grey pruinosity; tergites 2–6 each with a blackish brown medial spot and a pair of blackish brown triangular lateral spots. ***Male genitalia*** (Figs 27–31): syntergosternite 7+8 circular with 1 membranous triangular ventral process; epandrium rectangular in lateral view. Surstylus curved in lateral view, finger-like in posterior view. Hypandrium H-shaped. Pregonite slender, finger-like, postgonite slender coniform. Phallus knife-like in lateral view; phallapodeme about as long as phallus.

**Female**. Body length 4.0–4.2 mm; wing length 3.9–4.0 mm. Sternite VIII approximately as long as wide, with a rounded anterior margin. Sternite IX narrow, wider than long.

##### Distribution.

China (Sichuan, Chongqing).

##### Remarks.

The new species is somewhat similar to Homoneura (H.) subnubecula Sasakawa, 2001 in the wing pattern, but it differs in having a face with 1 brown median stripe, an arista with the longest ray shorter than 1/6 of the height of the first flagellomere; the mesonotum without irregular brown spot or stripe and the setulae and setae are without brown basal spot; the fore femur dark brown; the syntergosternite 7+8 circular with 1 ventral process; and the pregonite shorter than the phallus. In H. (H.) subnubecula, the fore femur is yellow, the syntergosternite is semicircular, and the pregonite is longer than the phallus (Sasakawa 2001: fig. 31).

It is also similar to Homoneura (H.) furcata sp. nov. based on the two round or hexagonal brown spots with hyaline rings between crossvein r-m and the apical spot on the wing, but in H. (H.) furcata sp. nov., syntergosternite 7+8 is semicircular, and the hypandrium is H-shaped, with a long, bifurcated ventral process.

## Discussion

The subgenus *Homoneura* Wulp comprises over 220 described species in China, which were classified into 21 species groups based on the characteristics of the male genitalia and patterning on the wing (Shi and Yang 2009, 2014). Phylogenetic studies by Kong et al. (2022) revealed that the subgenus *Homoneura* is polyphyletic. Subsequently, a controversial 12-species-group classification system was proposed by Yao et al. (2024) based on phylogenetic investigations of the subgenus *Homoneura*. However, this revised framework shows substantial inconsistencies with the established species-group delineations proposed by Shi and Yang (2009, 2014). Moreover, the proposed 12-group classification currently lacks sufficient phylogenetic evidence to substantiate its taxonomic delineations. Considering these significant limitations and the clear necessity for comprehensive phylogenetic studies to clarify infrageneric relationships within *Homoneura*, the present study adopts the well-established 21-species-group classification system.

**Figures 32–40.**
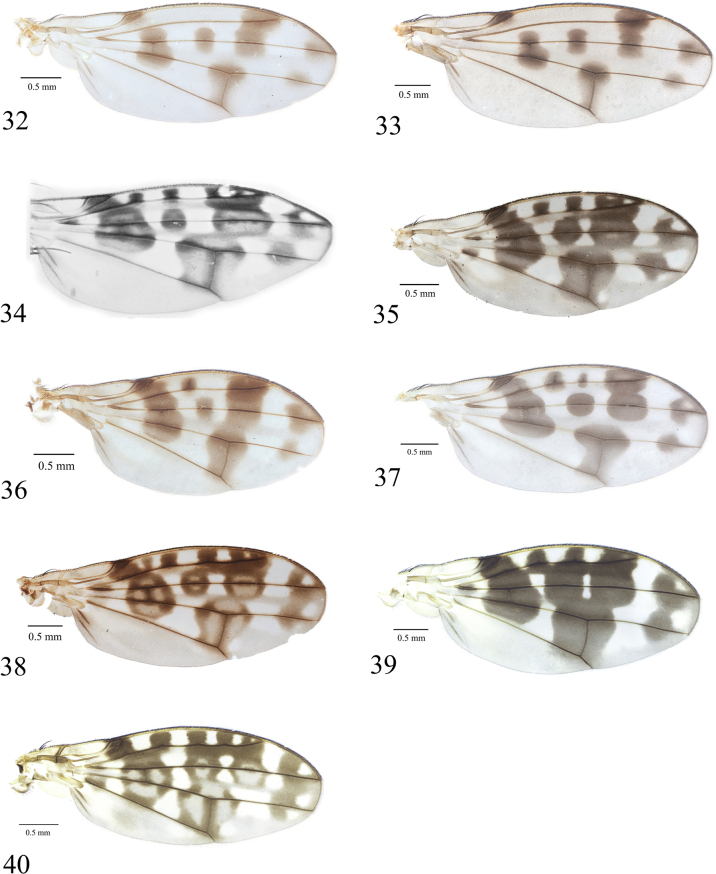
Wing. **32**. H. (H.) fengyangshanica Shi & Yang, 2014; **33**. H. (H.) dorsocuspidata Gao & Shi, 2019; **34**. H. (H.) apicitrunctata Gao, Shi & Han, 2016 (reproduced from Gao et al. 2016); **35**. H. (H.) concava Sasakawa, 2002; **36**. H. (H.) posterotricuspis Gao, Shi & Han, 2016; **37**. H. (H.) dorsacerba Gao, Shi & Han, 2016; **38**. H. (H.) furcata sp. nov.; **39**. H. (H.) lishuiensis sp. nov.; **40**. H. (H.) sexangula sp. nov.

The Homoneura (H.) singularis group is distinctly different from other species groups in wing pattern (Figs 32–40), having two brown spots present between r-m and the apical spot on R_4+5_ (in H. (H.) latifrons Malloch, several Japanese specimens have three brown spots between r-m and apical spot on R_4+5_; Sasakawa and Ikeuchi 1982), cell r_1_ is with or without spot, preapical or apical spots are present on R_2+3_ and M_1_, and there are stripe-like spots or clouds on r-m and dm-cu (Shi and Yang 2009, 2014). The three new species described herein exhibit remarkable congruence with the following four species in both wing pattern (spot number and position) and genitalic structure: H. (H.) concava Sasakawa, 2002; H. (H.) dorsacerba Gao, Shi & Han, 2016; H. (H.) apicitrunctata Gao, Shi & Han, 2016; H. (H.) posterotricuspis Gao, Shi & Han, 2016. The three species described in this paper are provisionally assigned to the *singularis* group, following Shi and Yang (2014) and Gao et al. (2016). There are now 21 species belonging to the *singularis* group in China (Becker 1895; Malloch 1927; Sasakawa and Ikeuchi 1985; Sasakawa 2001, 2002; Yang et al. 2002; Yang et al. 2003; Gao and Yang 2004; Shi and Yang 2014; Gao et al. 2016; Shen et al. 2018; Zhang et al. 2019).

## Supplementary Material

XML Treatment for Homoneura (Homoneura) furcata

XML Treatment for Homoneura (Homoneura) lishuiensis

XML Treatment for Homoneura (Homoneura) sexangula

## Additional information

### Use of AI

No use of AI was reported.

### Author ORCIDs

Peng-Yan You https://orcid.org/0000-0002-7485-8056

Xu-Long Chen https://orcid.org/0000-0003-0787-3333

Wen-Liang Li https://orcid.org/0000-0001-9019-1223

### Data availability

All of the data that support the findings of this study are available in the main text.
